# Supplementary material for: Gasdermin D promotes influenza virus-induced mortality through neutrophil amplification of inflammation
Source: Nat Commun. 2024 Mar 29;15:2751. doi: 10.1038/s41467-024-47067-0 (PMC10980740; doi:10.1038/s41467-024-47067-0)
Supplement: Supplementary file 1 — Supplementary Information [file 41467_2024_47067_MOESM1_ESM.pdf]

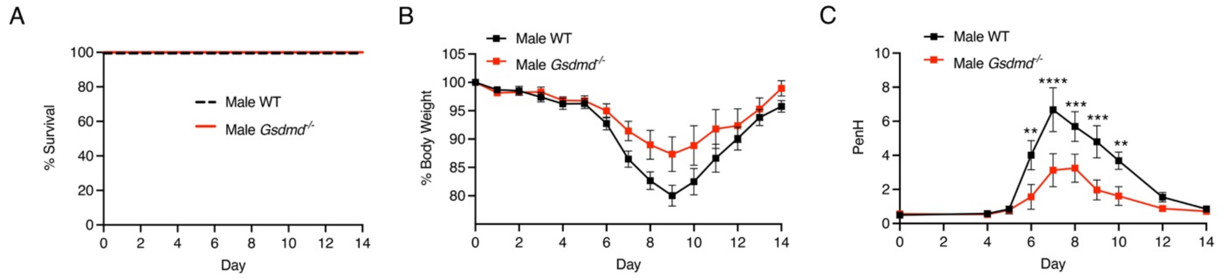

Supplementary Figure 1: Male *Gsdmd$^{-/-}$* mice experience decreased lung dysfunction in IAV infection. A-C Male WT and *Gsdmd$^{-/-}$* mice were intranasally infected with 50 TCID50 of IAV strain PR8. **A** Survival curve from 2 independent experiments with n=9 WT, n=11 *Gsdmd$^{-/-}$*. No fatalities were observed in either group. **B** Weight loss measurement following IAV infection (each dot is an average of individual mouse weights normalized to 100% relative to day 0, error bars indicate SEM, n=9 WT, n=11 *Gsdmd$^{-/-}$* from 2 independent experiments, statistical significance not achieved at any timepoint by two-way ANOVA with Bonferroni's Multiple Comparison's test). **C** PenH measurements (each dot is an average of individual mouse PenH values, error bars indicate SEM, n=9 WT, n=11 *Gsdmd$^{-/-}$* from 2 independent experiments), **p < 0.01, ***p < 0.001, ****p < 0.0001 by two-way ANOVA with Bonferroni's Multiple Comparison's test).

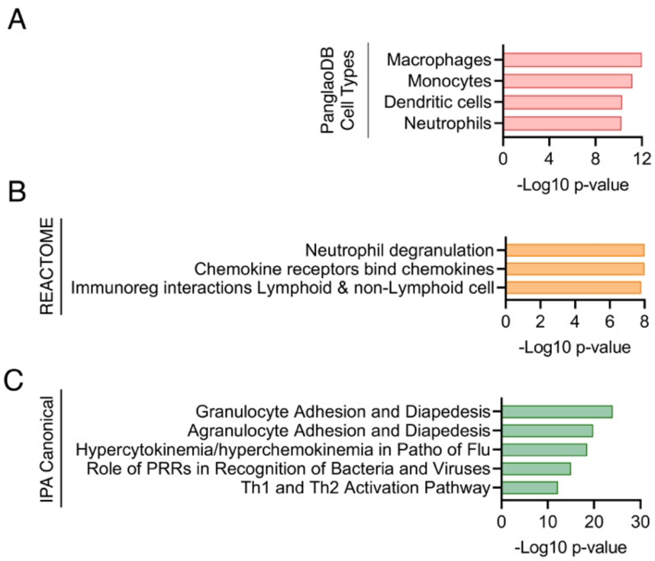

Supplementary Figure 2: Pathway analysis of gene signatures downregulated in *Gsdmd*$^{-/-}$ **versus WT mice. A-C** Differentially expressed genes in day 7 post infection *Gsdmd*$^{-/-}$ lungs versus WT (n=3 per group) were subjected to pathway and cell type analysis. **A** Downregualted genes in *Gsdmd*$^{-/-}$ versus WT lungs were subjected to PanglaoDB analysis and all significant associations with specific cell types are shown. **B** Significant REACTOME gene set enrichments ($p < 0.05$ by Gene Set Enrichment Analysis) for all downregulated genes in *Gsdmd*$^{-/-}$ versus WT lungs. **C** All differentially expressed genes were examined using Ingenuity Pathway Analysis and the top five most significant Canonical Pathways are shown.

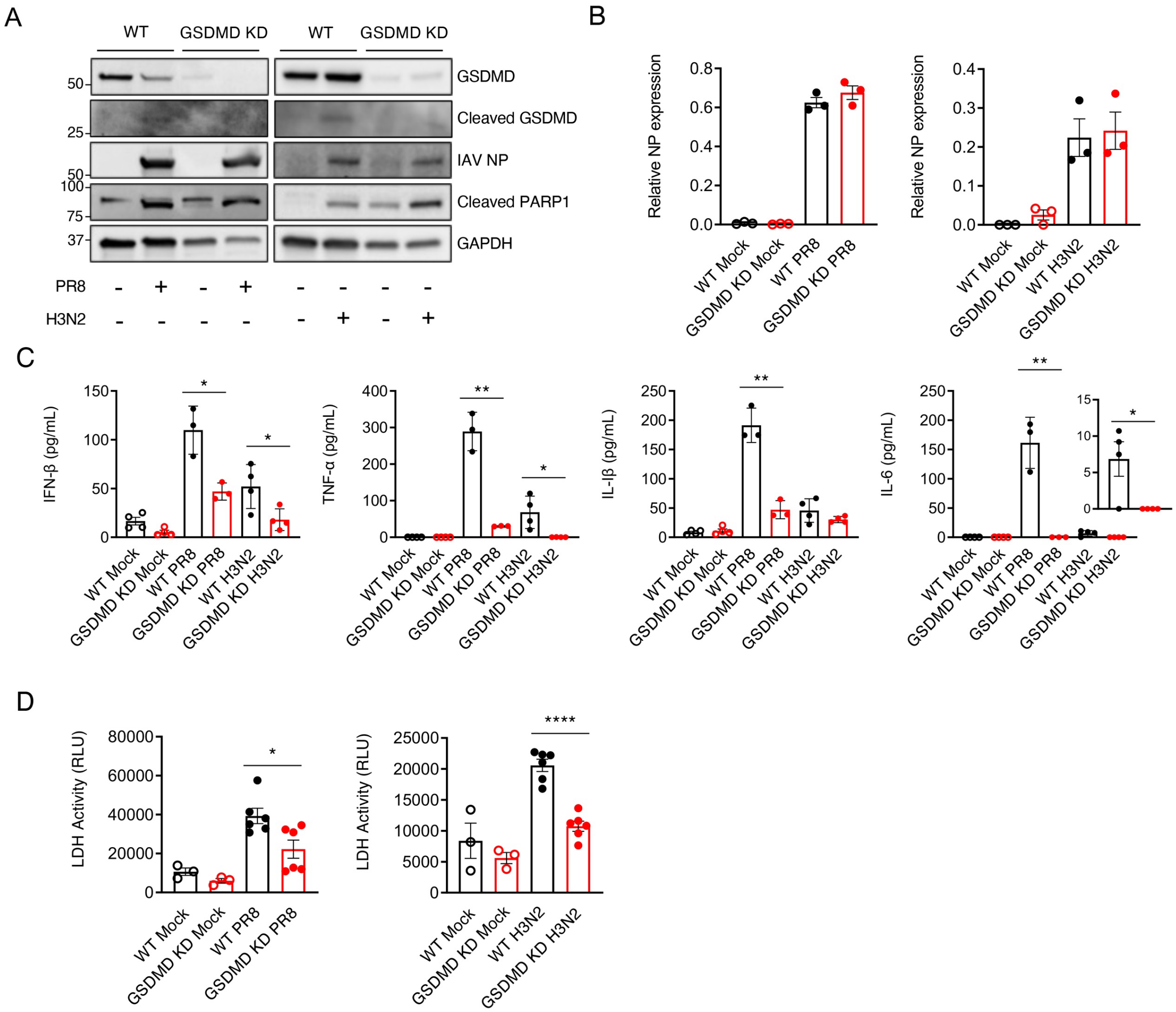

Supplementary Figure 3: Human GSDMD promotes secretion of pro-inflammatory cytokines in THP-1 macrophages *in vitro*. A Representative western blots for PMA-differentiated WT and GSDMD knockdown (KD) human THP-1 macrophages 48 hours post infection with PR8 or H3N2 at an MOI of 10. Results are representative of at least 3 experiments. **B** Densitometry quantification of NP levels relative to GAPDH as in **A** from 3 experiments. **C** ELISA quantification of IL-6, IFN-β, IL-1β, or TNF-α levels in supernatants from cells infected as in **A** (each dot represents an average result from 1 of at least 3 experiments, $^*p < 0.05$, $^{**}p < 0.01$ by two-way ANOVA with Tukey's multiple comparisons test). **D** Relative extracellular lactate dehydrogenase (LDH) levels quantified in supernatants of infected cells as in **A** via luminescence-based assay (RLU, relative light units, n=3 mocks from 1 experiment and n=6 infected samples from 2 experiments, $^*p < 0.05$, $^{****}p < 0.0001$ by two-way ANOVA with Tukey's multiple comparisons test, only comparisons of interest are shown).

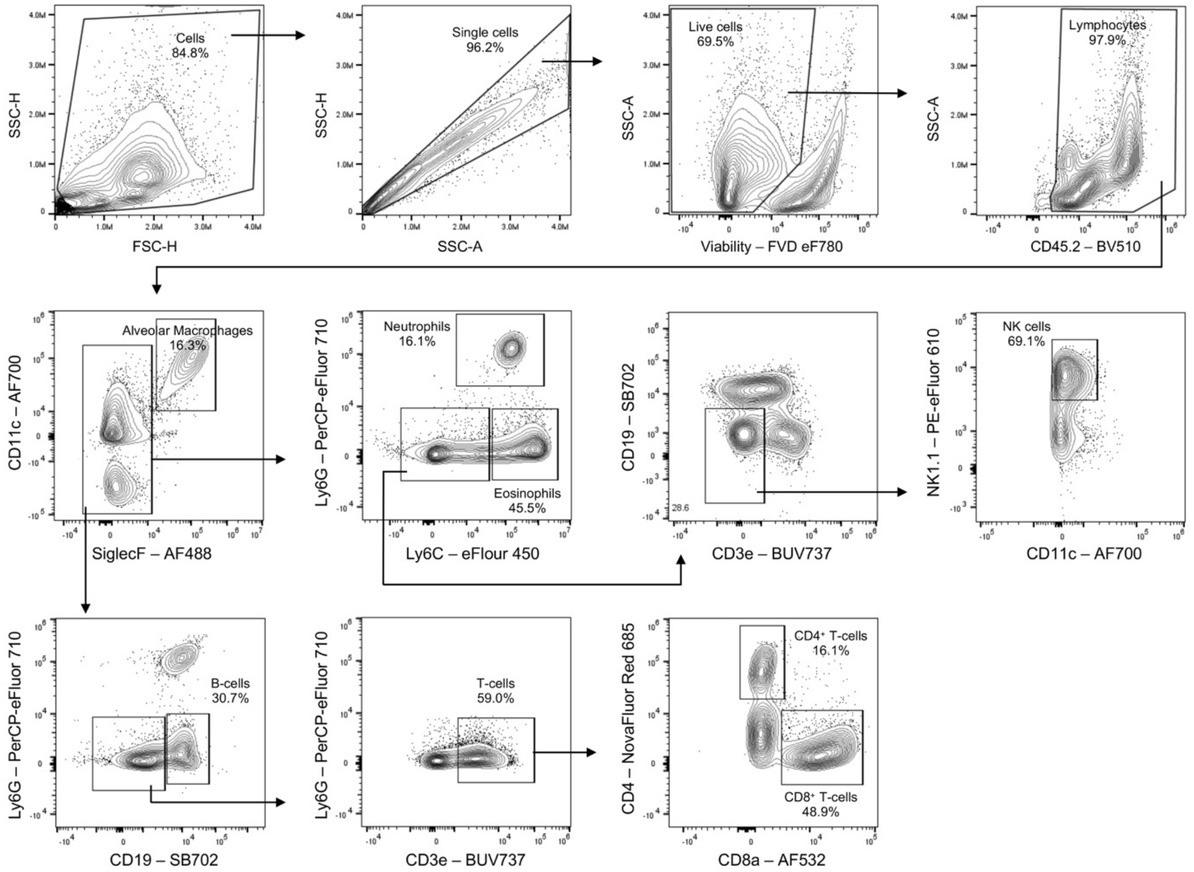

Supplementary Figure 4: Gating strategy for quantifying immune cell recruitment to mouse lungs during IAV infection. In addition to Fixable Viability Dye-eFluor 780, antibody target-fluorophore conjugates include: CD45.2-BV510, CD11c-AF700, SiglecF-AF488, Ly6C-eFluor 450, Ly6G-PerCP-eFluor 710, CD19-SB702, CD3e-BUV737, NK1.1-PE-eFluor 780, CD4-NovaFluor Red 685, CD8a-SB780.

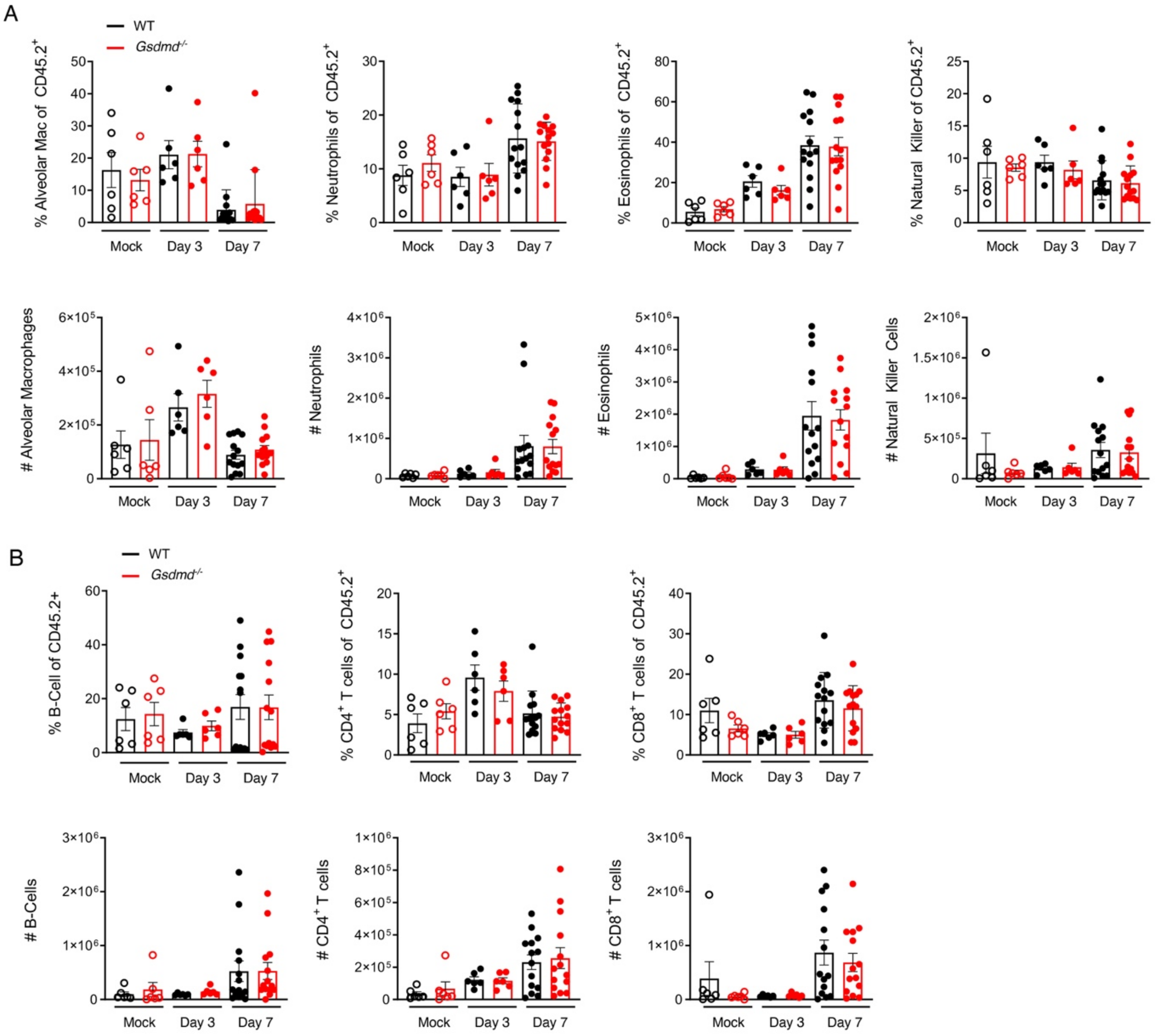

Supplementary Figure 5: GSDMD does not affect immune cell recruitment to the lung during IAV infection. A-B WT and *Gsdmd*$^{-/-}$ mice were infected with 50 TCID50 of IAV strain PR8. Analysis was done on day 3 or 7 post infection using the flow cytometry gating strategy show in Supplementary Figure 4. Percentage (%) of the indicated cell type relative to all CD45.2^{+} immune cells and total cell numbers (#) are graphed. Data for innate immune cells (**A**) and adaptive immune cells (**B**) are pooled from two separate experiments for mocks and day 3 animals (n=6) and from 3 separate experiments for day 7 animals (n=14). Each dot represents an individual mouse and error bars indicate SEM. No significant differences between WT and *Gsdmd*$^{-/-}$ samples for mocks or on day 3 or day 7 were detected by ANOVA with Tukey's multiple comparisons test for any cell type % or #. Neutrophil # data is repeated from **Fig 5C** in the main text.

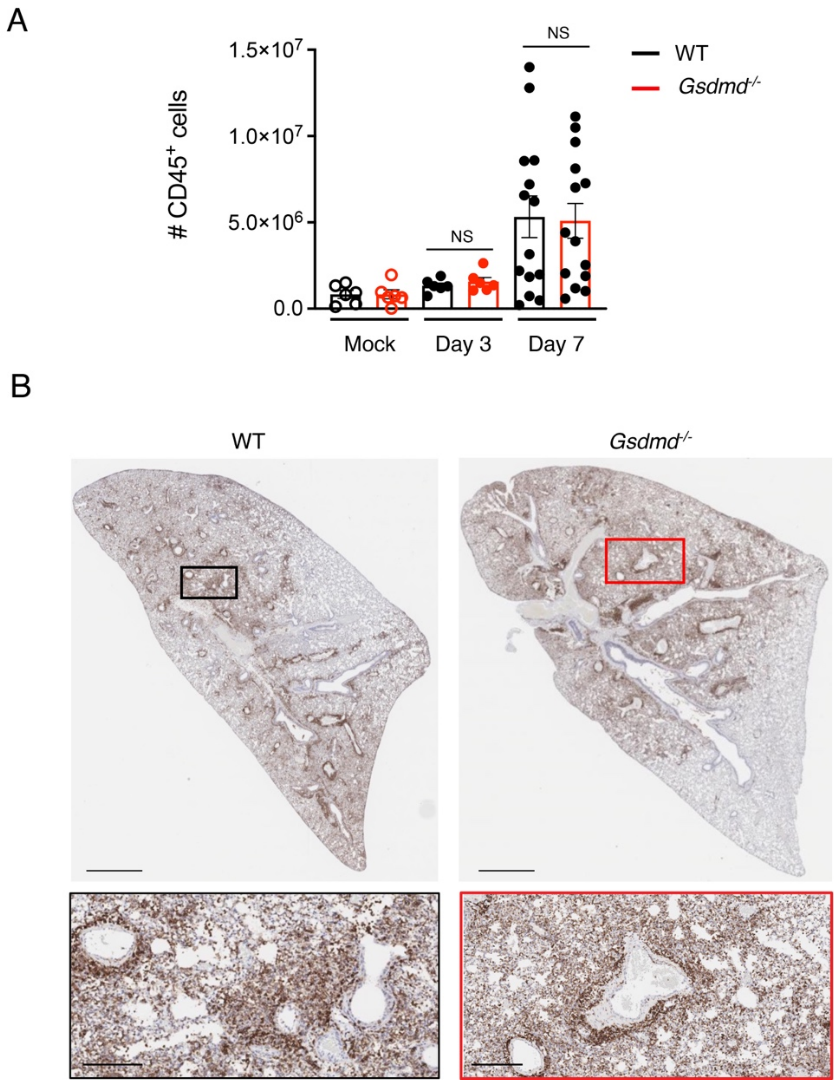

Supplementary Figure 6: CD45+ immune cells are present in IAV-infected lungs independent of GSDMD. WT and *Gsdmd*$^{-/-}$ mice were intranasally infected with 50 TCID50 of IAV strain PR8. **A** Flow Cytometry analysis was done on day 3 or 7 post infection gating strategy show in Supplementary Figure 4. Cell numbers (#) are graphed. Data are pooled from two separate experiments for mocks and day 3 animals (n=6) and from 3 separate experiments for day 7 animals (n=14). Each dot represents an individual mouse and error bars indicate SEM. No significant differences between WT and *Gsdmd*$^{-/-}$ samples for mocks or on day 3 or day 7 were detected by ANOVA with Tukey's multiple comparisons test. **B** Representative anti-CD45 stained (brown) images for WT and *Gsdmd*$^{-/-}$ mouse lungs at day 7 post-infection. Red and blue boxed regions are magnified below the full lung sections and highlight cell infiltration in both WT and *Gsdmd*$^{-/-}$ samples. Scale bars represent 2 mm for the full lung section and 250 μm for the magnified region.

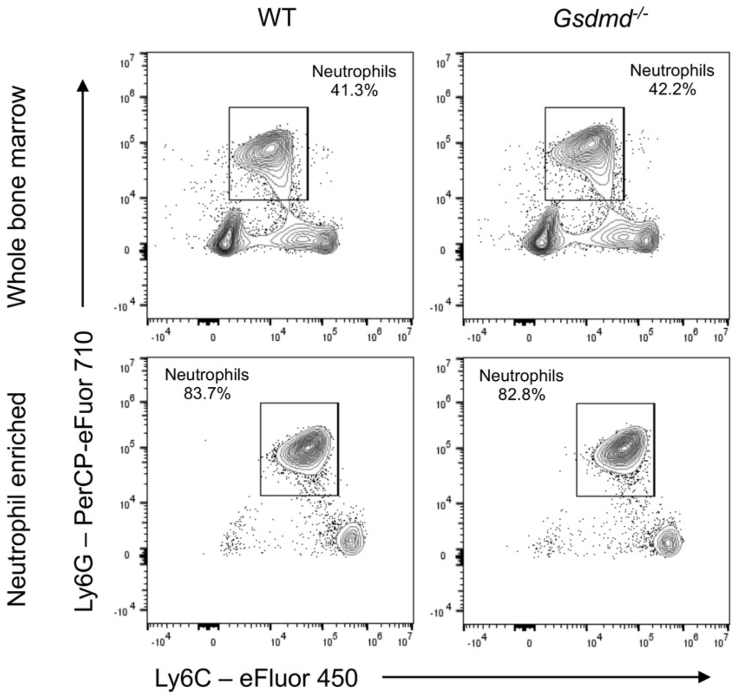

Supplementary Figure 7: Neutrophil enrichment from WT and *Gsdmd$^{-/-}$* mouse bone marrow. Representative flow cytometry dot plots showing neutrophil enrichment for experiments in **Fig5E-G**. Upstream gating performed as in **Supplementary Figure 4**.

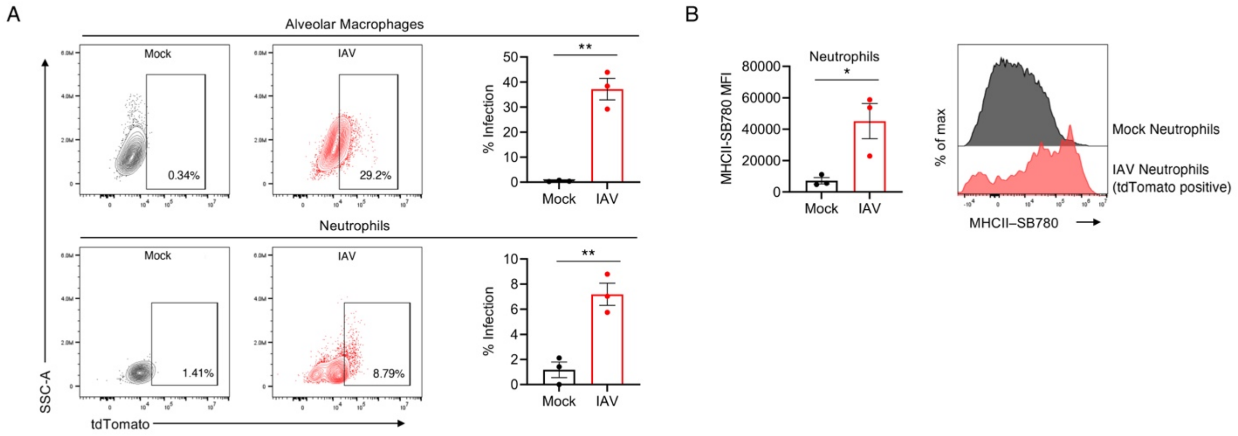

Supplementary Figure 8: Neutrophils are infected by IAV *in vivo* and upregulate MHCII. A-B Floxed allele tdTomato reporter mice were intranasally infected with 250 TCID50 of PR8-Cre and sacrificed on day 7 post infection for analysis of tdTomato^{+} immune cells in the lungs via flow cytometry. **A** Representative flow plots showing levels of infection in macrophages, neutrophils. Upstream gating performed as in **Supplementary Figure 4**. Data quantified in bar graphs to the right (n = 3 mock and IAV-infected mice, *p < 0.05, unpaired t-test). **B** Representative histogram showing MHCII mean fluorescence intensity for neutrophils from mock animals and for tdTomato positive neutrophils from IAV-infected animals. Mean fluorescence intensity (MFI) for MHCII detection for these two populations is quantified in the bar graph (n = 3 mock and infected mice, *p < 0.05, unpaired t-test).

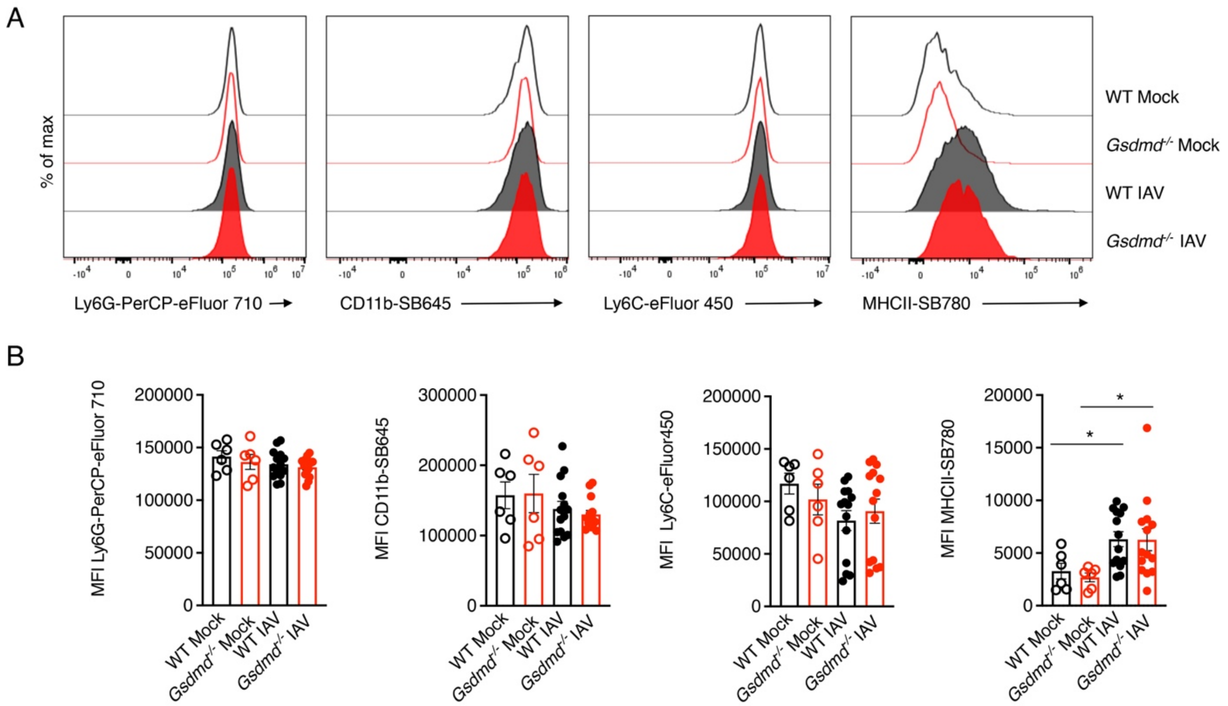

Supplementary Figure 9: Analysis of neutrophil levels of surface markers following IAV infection. A-B Further analysis of neutrophils from day 7 flow cytometry data in **Fig5C**. **A** Representative histograms showing mean fluorescence of neutrophil markers in WT and *Gsdmd*$^{-/-}$ mice. **B** Quantification of mean fluorescence intensity (MFI) for the indicated markers as in **A** (n=6 mocks and n=14 infected mice, error bars indicate SEM, statistical significance was not achieved for any comparisons by ANOVA followed by Tukey's multiple comparisons test, statistical significance ($p < 0.05$) was observed by unpaired t-test comparing MHCII MFI for WT Mock versus WT IAV and *Gsdmd*$^{-/-}$ mock versus *Gsdmd*$^{-/-}$ IAV).

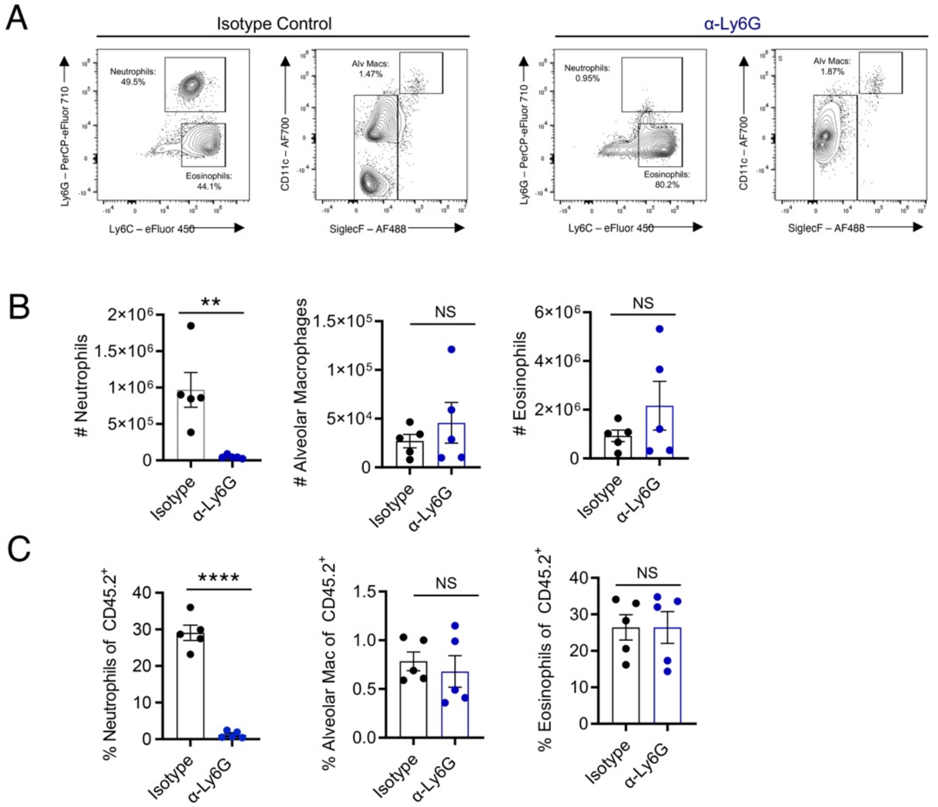

Supplementary Figure 10: Anti-Ly6G antibody treatment selectively depletes neutrophils. A-C WT mice were infected as in **Fig 6A**. **A** Representative flow cytometry plots showing neutrophil, eosinophil, and alveolar macrophage gating from lungs on day 5 post infection. Antibody target-fluorophore combinations and upstream gating are as in **Supplementary Figure 4**. **B** Neutrophil, eosinophil, and alveolar macrophage total number (#) in the lung on day 5 post infection of n=5 mice (**$p < 0.01$, NS, not significant, unpaired t-test). Neutrophil # data is repeated from **Fig 6B** in main text. **C** Neutrophil, eosinophil, and alveolar macrophage percentage of all $CD45.2^+$ cells in the lung on day 5 post infection of n=5 mice (****$p < 0.0001$, NS, not significant, unpaired t-test). **B,C** Each dot represents measurements from an individual mouse, error bars indicate SEM.

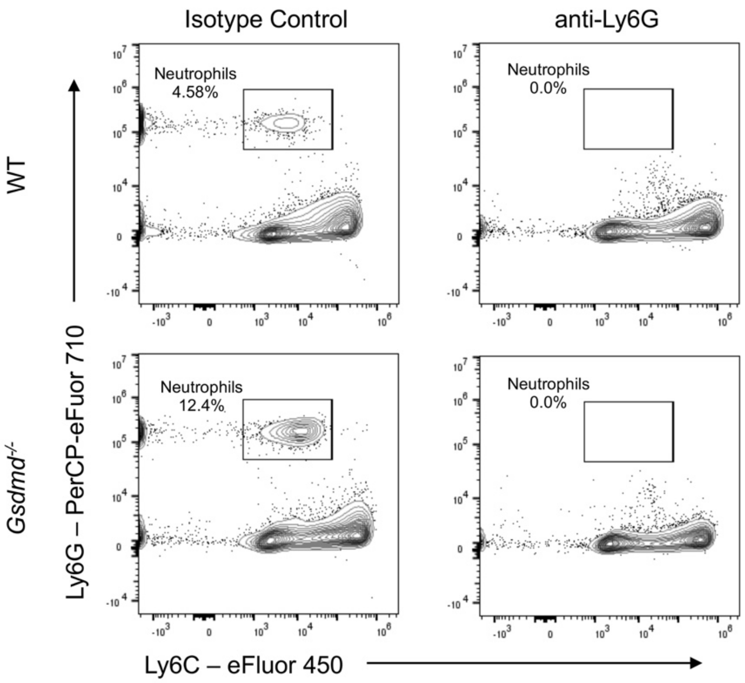

Supplementary Figure 11: Anti-Ly6G antibody treatment depletes neutrophils in *Gsdmd*$^{-/-}$ mice as expected. Representative flow cytometry dot plots showing neutrophil gating of single-cell suspensions from the lungs of mice on day 5 post infection with 50 TCID50 IAV strain PR8 as in **Fig 6G**. These plots are representative of two mice randomly chosen to be sacrificed to confirm neutrophil depletion. Upstream gating performed as in **Supplementary Figure 4**.

Full blots for Supplementary Figure 3A, left

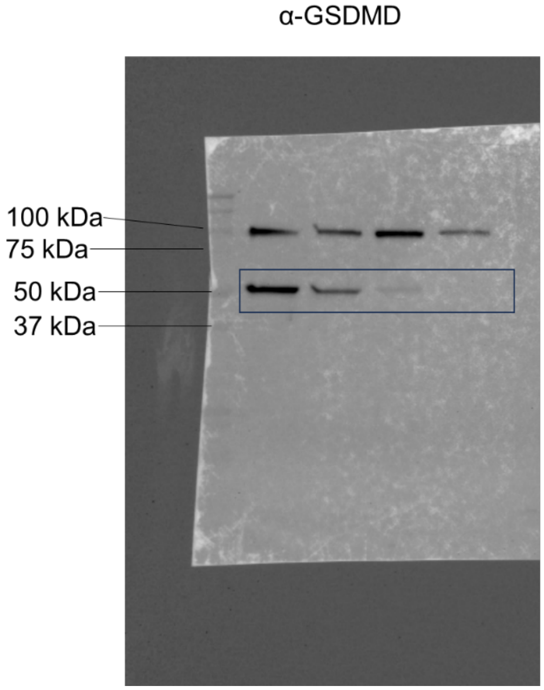

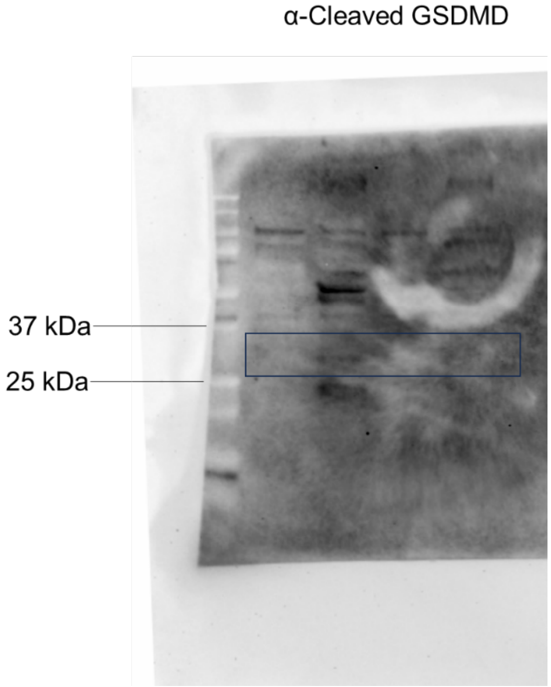

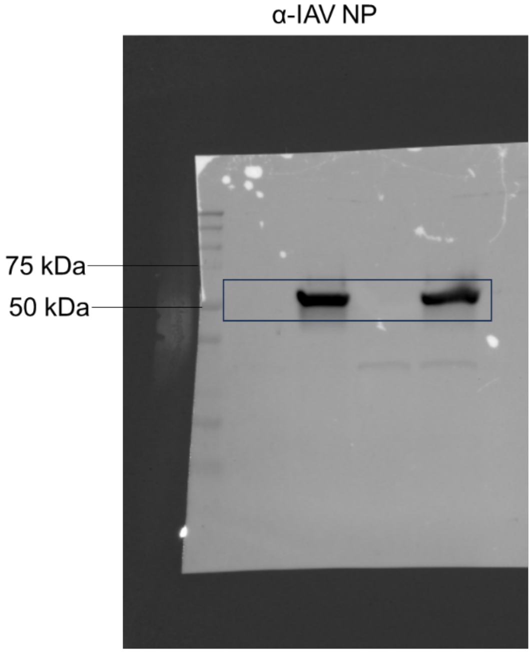

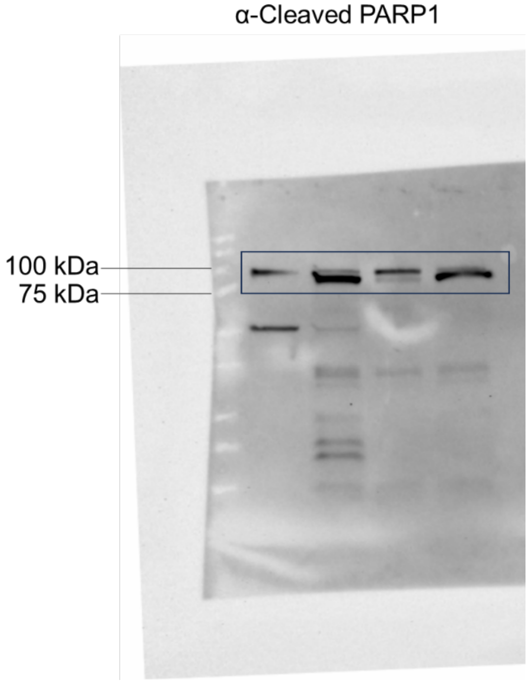

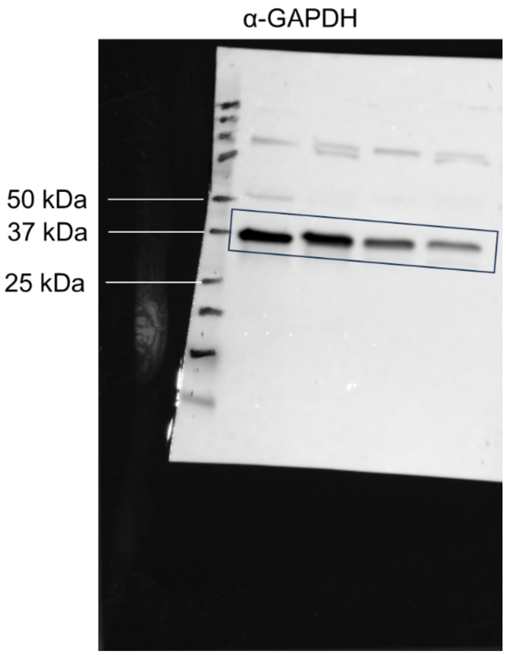

Full blots for Supplementary Figure 3A, right

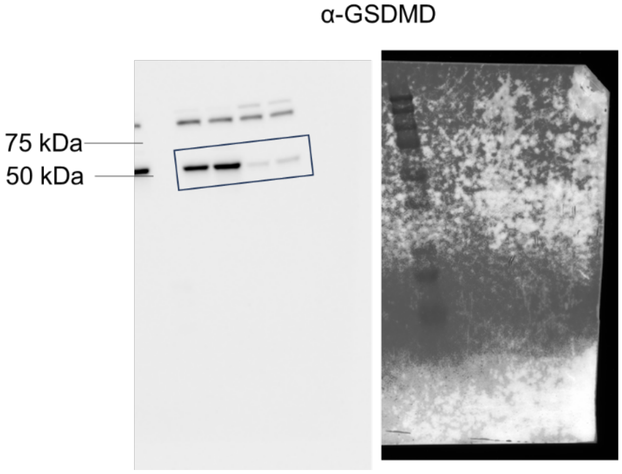

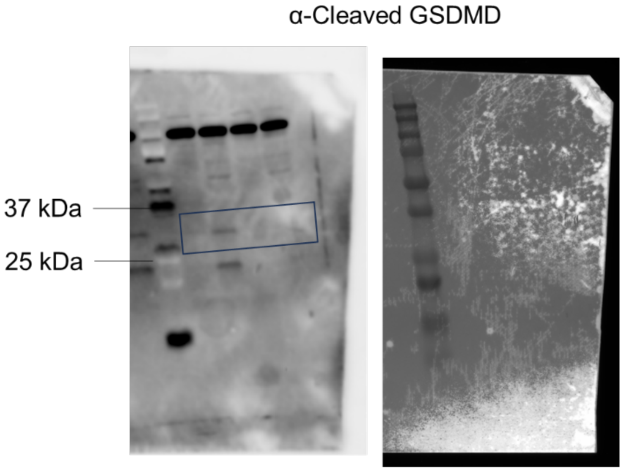

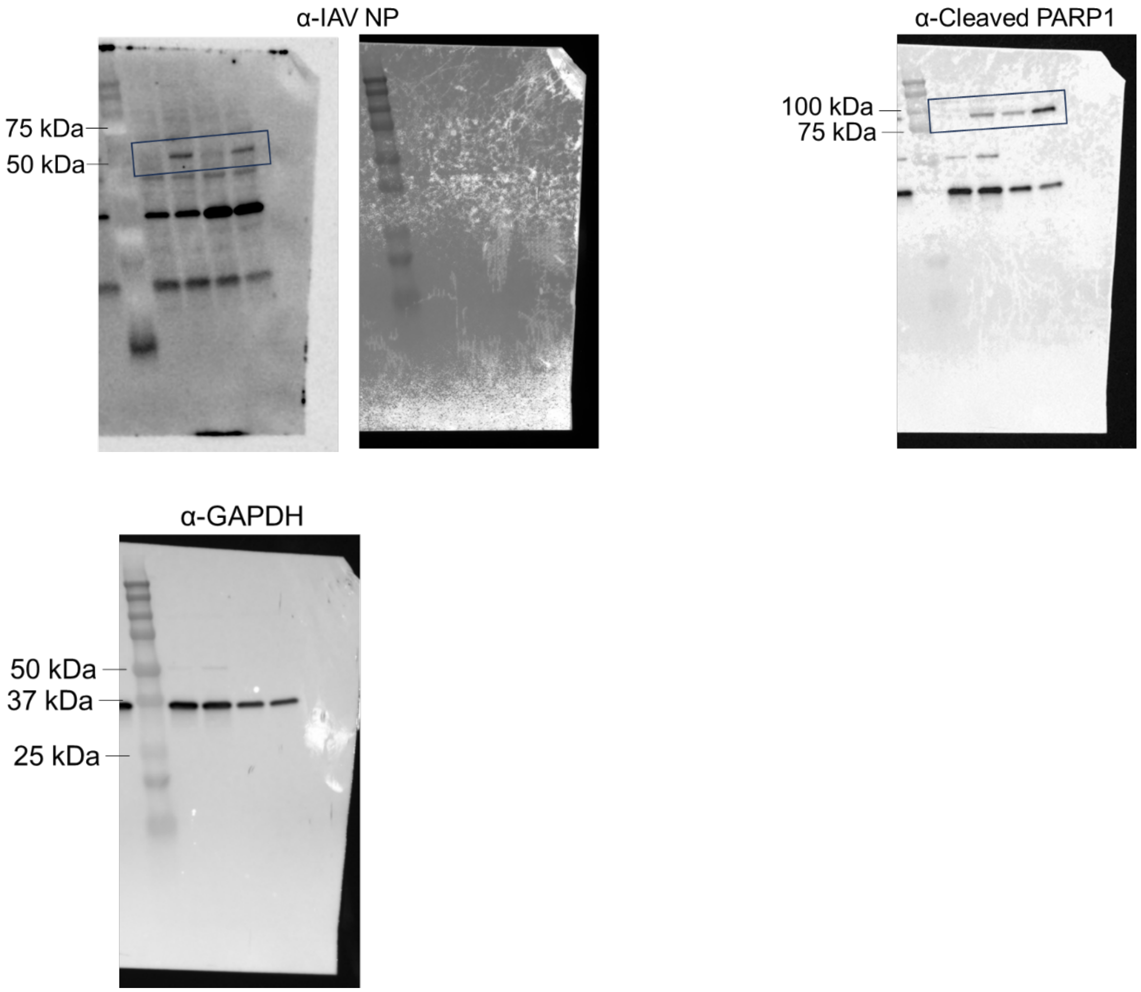
α-IAV NP
75 kDa
50 kDa
α-Cleaved PARP1
100 kDa
75 kDa
α-GAPDH
50 kDa
37 kDa
25 kDa
