# Supplementary material for: Gasdermin D promotes influenza virus-induced mortality through neutrophil amplification of inflammation
Source: Nat Commun. 2024 Mar 29;15:2751. doi: 10.1038/s41467-024-47067-0 (PMC10980740; doi:10.1038/s41467-024-47067-0)
Supplement: Supplementary file 3 — Description of Additional Supplementary Files [file 41467_2024_47067_MOESM3_ESM.pdf]

Description of Additional Supplementary Files

Supplementary Data 1: All differentially expressed genes (lfc |1.5|, adj p-value <0.01) comparing WT versus *Gsdmd-/-* lungs at day 7 post infection with 50 TCID50 IAV strain PR8.

Supplementary Data 2:

A: Differentially expressed genes associated with Defense to Virus related to Main Text Figure 4A.

B: Differentially expressed genes associated with Neutrophil Chemotaxis related to Main Text Figure 4B.

Supplementary Data 3: Primer sequences used for qRT-PCR.
