# Supplementary material for: Gasdermin D promotes influenza virus-induced mortality through neutrophil amplification of inflammation
Source: Nat Commun. 2024 Mar 29;15:2751. doi: 10.1038/s41467-024-47067-0 (PMC10980740; doi:10.1038/s41467-024-47067-0)
Supplement: Supplementary file 7 — Reporting Summary [file 41467_2024_47067_MOESM7_ESM.pdf]

nature portfolio

Corresponding author(s): Jacob S. Yount

Last updated by author(s): March 1, 2024

Reporting Summary

Nature Portfolio wishes to improve the reproducibility of the work that we publish. This form provides structure for consistency and transparency in reporting. For further information on Nature Portfolio policies, see our Editorial Policies and the Editorial Policy Checklist.

Statistics

For all statistical analyses, confirm that the following items are present in the figure legend, table legend, main text, or Methods section.

n/a	Confirmed	
☐	☒	The exact sample size (*n*) for each experimental group/condition, given as a discrete number and unit of measurement
☐	☒	A statement on whether measurements were taken from distinct samples or whether the same sample was measured repeatedly
☐	☒	The statistical test(s) used AND whether they are one- or two-sided<br>*Only common tests should be described solely by name; describe more complex techniques in the Methods section.*
☒	☐	A description of all covariates tested
☐	☒	A description of any assumptions or corrections, such as tests of normality and adjustment for multiple comparisons
☐	☒	A full description of the statistical parameters including central tendency (e.g. means) or other basic estimates (e.g. regression coefficient) AND variation (e.g. standard deviation) or associated estimates of uncertainty (e.g. confidence intervals)
☐	☒	For null hypothesis testing, the test statistic (e.g. *F*, *t*, *r*) with confidence intervals, effect sizes, degrees of freedom and *P* value noted<br>*Give P values as exact values whenever suitable.*
☒	☐	For Bayesian analysis, information on the choice of priors and Markov chain Monte Carlo settings
☒	☐	For hierarchical and complex designs, identification of the appropriate level for tests and full reporting of outcomes
☒	☐	Estimates of effect sizes (e.g. Cohen's *d*, Pearson's *r*), indicating how they were calculated

Our web collection on statistics for biologists contains articles on many of the points above.

Software and code

Policy information about availability of computer code

Data collection	No proprietary or non-commercial/non-publicly available software was used in this manuscript.
Data analysis	No proprietary or non-commercial/non-publicly available software was used in this manuscript. FlowJo (v10.8.0) was used for flow cytometric analysis. DESeq2 was used for determination of differentially expressed genes and p values (Love, M. I., Huber, W. & Anders, S. Moderated estimation of fold change and dispersion for RNA-seq data with DESeq2. Genome Biol. 15, 550 (2014)). GraphPad (9.0) software was used for generating graphs and statistical calculations. Pathway analysis was performed with Enrichr (https://maayanlab.cloud/Enrichr/). Gene Set Enrichment Analysis was performed using the Broad Institute's GSEA software version 4.3.2 with Gene Ontology Biological Pathways terms or REACTOME terms. Network analysis of GSEA results was performed with Cytoscape version 3.9.1. Generation of figures for RNA-sequencing results was performed using R version 2023.03.0+386 or GraphPad Prism version 9. For Figures 4A-B, murine gene sets for indicated GO terms were downloaded from AmiGO 2 (https://amigo.geneontology.org/amigo). For Figure 5A, murine gene sets for indicated KEGG terms were downloaded from GenomeNet (https://www.genome.jp/kegg/kegg2.html).

For manuscripts utilizing custom algorithms or software that are central to the research but not yet described in published literature, software must be made available to editors and reviewers. We strongly encourage code deposition in a community repository (e.g. GitHub). See the Nature Portfolio guidelines for submitting code & software for further information.

Data

Policy information about availability of data

All manuscripts must include a data availability statement. This statement should provide the following information, where applicable:

- Accession codes, unique identifiers, or web links for publicly available datasets
- A description of any restrictions on data availability
- For clinical datasets or third party data, please ensure that the statement adheres to our policy

RNAseq data was deposited to the NCBI GEO database under accession number GSE230656. Gene Set Enrichment Analysis was performed using the Broad Institute's GSEA software version 4.3.2 with Gene Ontology Biological Pathways terms or REACTOME terms. Network analysis of GSEA results was performed with Cytoscape version 3.9.1. For Figures 4A-B, murine gene sets for indicated GO terms were downloaded from AmiGO 2 (https://amigo.geneontology.org/amigo). For Figure 5A, murine gene sets for indicated KEGG terms were downloaded from GenomeNet (https://www.genome.jp/kegg/kegg2.html). Other source data contained in the manuscript are available in the Source Data file.

Research involving human participants, their data, or biological material

Policy information about studies with human participants or human data. See also policy information about sex, gender (identity/presentation), and sexual orientation and race, ethnicity and racism.

Reporting on sex and gender	N/A
Reporting on race, ethnicity, or other socially relevant groupings	N/A
Population characteristics	N/A
Recruitment	N/A
Ethics oversight	N/A

Note that full information on the approval of the study protocol must also be provided in the manuscript.

Field-specific reporting

Please select the one below that is the best fit for your research. If you are not sure, read the appropriate sections before making your selection.

[x] Life sciences [ ] Behavioural & social sciences [ ] Ecological, evolutionary & environmental sciences

For a reference copy of the document with all sections, see nature.com/documents/nr-reporting-summary-flat.pdf

Life sciences study design

All studies must disclose on these points even when the disclosure is negative.

Sample size	A minimum of n=3 was chosen because it is the minimal replicate number sufficient to perform statistical analysis by t-test or ANOVA/multiple comparisons test.
Data exclusions	No exclusions.
Replication	RNAseq analysis was performed on n=3 per group. All other experiments were performed with an n greater than or equal to three animals per group for each experiment. All data shown in the manuscript were reproducible.
Randomization	Groups were randomized prior to infection or treatments.
Blinding	Blinding was not possible in this study because experimenters carried out treatments, measurements, and analysis.

Reporting for specific materials, systems and methods

We require information from authors about some types of materials, experimental systems and methods used in many studies. Here, indicate whether each material, system or method listed is relevant to your study. If you are not sure if a list item applies to your research, read the appropriate section before selecting a response.

Materials & experimental systems

n/a	Involved in the study
☐	☒ Antibodies
☐	☒ Eukaryotic cell lines
☒	☐ Palaeontology and archaeology
☐	☒ Animals and other organisms
☒	☐ Clinical data
☒	☐ Dual use research of concern
☒	☐ Plants

Methods

n/a	Involved in the study
☒	☐ ChIP-seq
☐	☒ Flow cytometry
☒	☐ MRI-based neuroimaging

Antibodies

Antibodies used

Western blotting antibodies:
(Supplier, name, clone, dilution, reference number)
Abcam, anti-GSDMD, clone EPR20859, 1:1000, ref ab219800
Abcam, anti-Actin, clone ACTN05 (C4), 1:1000, ref ab3280
Abcam, Recombinant anti-GSDMD, clone EPR19829, 1:1000, ref ab210070
ThermoFisher, anti-GAPDH, clone ZG003, 1:1000, ref 39-8600
Cell Signaling Technologies HRP-conjugated anti-Rabbit IgG, 1:10000, ref 7074
Cell Signaling Technologies anti-Cleaved PARP (Asp214), clone D64E10, 1:1000, ref 5625
BEI Resources anti-Influenza NP A/CA/04/09 (H1N1) pdm, clone 2F4, 1:1000, ref NR-19868
Cell Signaling Technologies anti-GAPDH, clone 14C10, 1:1000, ref 2118
Cell Signaling Technologies anti-Cleaved GSDMD (Asp275), clone E7H9G, 1:1000, ref 36425
Jackson ImmunoResearch Peroxidase AffiniPure Donkey anti-Mouse IgG (H+L), 1:10000, ref 715-035-150
Jackson ImmunoResearch Peroxidase AffiniPure Donkey anti-Rabbit IgG (H+L), 1:10000, ref 711-035-152

Invitrogen flow cytometry antibodies:
(Target, clone, fluorophore, dilution, reference number)
Ly6G, clone 1A8-Ly6g, PerCP-eFluor 710, 1:133, ref 46-9668-82
CD11b, clone M1/70, Super Bright 645, 1:333, ref 64-0112-82
CD103, clone 2E7, Super Bright 600, 1:333, ref 63-1031-82
CD45R, clone RA3-6B2, PE, 1:333, ref 12-0452-82
MHC Class II, clone M5/114.15.2, Super Bright 780, 1:500, ref 78-5321-82
CD8a, clone 53-6.7, Alexa Fluor 532, 1:333, ref 58-0081-80
CD4, clone GK1.5, NovaFluor Red 685, 1:333, ref M001T02R02
CD3e, clone 17A2, Brilliant Ultra Violet 737, 1:200, ref 367-0032-82
CD69, clone H1.2F3, PE-Cyanine7, 1:133, ref 25-0691-82
CD19, clone eBio1D3 (1D3), Super Bright 702, 1:333, ref 67-0193-82
NK1.1, clone PK136, PE-eFluor 610, 1:200, ref 61-5941-82
Ly6C, clone HK1.4, eFluor 450, 1:200, ref 48-5932-82

Biolegend flow cytometry antibodies:
(Target, clone, fluorophore, dilution, reference number)
CD45.2, clone 104, Brilliant Violet 510, 1:333, ref 109838
CD11c, clone N418, Alexa Fluor 700, 1:133, ref 117320
Siglec-F, clone S17007L, Alexa Fluor 488, 1:100, ref 155524

In vivo depletion antibodies:
(Supplier, name/target, clone, amount injected, reference number)
Bio X Cell, InVivoMAb anti-mouse Ly6G, clone 1A8, 0.5 mg/mouse on day 3 post infection and 0.2 mg/mouse on days 4-8 post infection, ref BE0075-1
Bio X Cell, InVivoMAb rat IgG2a isotype control anti-trinitrophenol, 0.5 mg/mouse on day 3 post infection and 0.2 mg/mouse on days 4-8 post infection, clone 2A3, ref BE0089

Validation

All primary western blotting antibodies were used at a concentration of 1:1000. Antibodies were validated by identification of specific signal at correct molecular weight (kDa). Mouse GSDMD antibody was also confirmed to be specific using GSDMD knockout mouse lungs. Anti-nucleoprotein antibody was also validated using non-infected cells compared to infected cells. Commercially available flow cytometry antibodies were utilized at the concentration specified on the manufacturer's website. Each antibody was validated using appropriate FMO/isotype controls for flow cytometry where applicable. Commercially available in-vivo depletion antibodies were used at the concentration shown to achieve effective depletion in the biomedical literature, including the following references: Moynihan, KD, et al, Nature Medicine, 2016; Conde, P, et al., Immunity, 2015; Griseri, T, et al., Immunity, 2015; Coffelt, SB, et al., Nature, 2015; Brandes, M, et al., Cell, 2013. Depletion antibodies were validated through identification of specific cell type depletion via flow cytometry.

Antibody validation statements:
Abcam: “Antibodies are validated in western blot using lysates from cells or tissues that we have identified to express the protein of interest. Once we have determined the right lysates to use, western blots are run and the band size is checked for the expected molecular weight. We will always run several controls in the same western blot experiment, including positive lysate and negative lysate. When possible, we also include knock-out (KO) cell lines as a true negative control for our western blots. We are always increasing the number of KO-validated antibodies we provide. In addition, we run old stock alongside our new stock. If we know the old stock works well, this also acts as a suitable positive control. If the western blot result gives a clear clean band and we are happy with the result from the control lanes, these antibodies will be passed and added to the catalog.” (https://www.abcam.com/primary-antibodies/how-we-validate-our-antibodies)
Thermo Fisher (including Invitrogen): “At Thermo Fisher Scientific, we help provide confidence in selecting the right antibody for the intended research needs to enable researchers to successfully reproduce and confirm experimental results. This means that an antibody must be specific and selective within the context it is being used. To achieve this, we use a comprehensive approach to antibody verification that is tailored to the antibody target and the relevant application. Our advanced verification occurs in addition to standard antibody testing that happens during manufacturing.” (https://www.thermofisher.com/us/en/home/life-science/antibodies/invitrogen-antibody-validation.html)

Cell Signaling Technologies: “To ensure our antibodies will work in your experiment, we adhere to the Hallmarks of Antibody Validation™, six complementary strategies that can be used to determine the functionality, specificity, and sensitivity of an antibody in any given assay. CST adapted the work by Uhlen, et. al., (“A Proposal for Validation of Antibodies.” Nature Methods (2016)) to build the Hallmarks of Antibody Validation, based on our decades of experience as an antibody manufacturer and our dedication to reproducible science. We guarantee that our antibodies are fit for purpose by carefully tailoring the combination of validation strategies applied to each product. This means customizing our validation process according to the biological role of the target, while considering the sensitivity requirements of the downstream assay, the availability of appropriate testing models, and the relevance of each method to target investigation.” (https://www.cellsignal.com/about-us/cst-antibody-validation-principles)
Jackson ImmunoResearch Donkey Anti-Mouse IgG: ”Based on immunoelectrophoresis and/or ELISA, the antibody reacts with whole molecule mouse IgG. It also reacts with the light chains of other mouse immunoglobulins. No antibody was detected against non-immunoglobulin serum proteins. The antibody has been tested by ELISA and/or solid-phase adsorbed to ensure minimal cross-reaction with bovine, chicken, goat, guinea pig, syrian hamster, horse, human, rabbit and sheep serum proteins, but it may cross-react with immunoglobulins from other species.” (https://www.jacksonimmuno.com/catalog/products/715-035-150)

Jackson ImmunoResearch Donkey Anti-Rabbit IgG: ”Based on immunoelectrophoresis and/or ELISA, the antibody reacts with whole molecule rabbit IgG. It also reacts with the light chains of other rabbit immunoglobulins. No antibody was detected against non-immunoglobulin serum proteins. The antibody has been tested by ELISA and/or solid-phase adsorbed to ensure minimal cross-reaction with bovine, chicken, goat, guinea pig, syrian hamster, horse, human, mouse, rat and sheep serum proteins, but it may cross-react with immunoglobulins from other species.” (https://www.jacksonimmuno.com/catalog/products/711-035-152)

BioLegend: “BioLegend spends a considerable effort in developing and creating new reagents for research. We produce a variety of recombinant proteins, antibodies, immunoassays, and multiomics tools. The below example will focus on our stringency in creating new monoclonal antibodies produced from hybridomas: Clones of these hybridomas are carefully selected based on a number of criteria including robust growth and efficient production of a single clone of antibody that is specific to the intended target. The best clones move on to applications testing. The steps include: immunogen design and construction, immunization of host animal, hybridoma creation, ELISA or application-specific screening of antibodies from clones, application testing, including WB, ELISA, ChIP, IF, IHC, or biofunctional assays. Antibody clones are then tested in a variety of assays to see which applications they are suited for. As an example, clone 13A3-1 for phosphorylated STAT3 (Tyr705) demonstrated excellent performance in flow cytometry, western blot, and chromatin immunoprecipitation. Thus, the clone cross-validates itself by demonstrating functionality across orthogonal testing methods. Additionally, the biological induction of the phosphorylated state using IL-6 further validates the specificity of the antibody.” (https://www.biolegend.com/en-ie/quality/product-development)

Bio X Cell (InVivoMAb): “We utilize a library of recombinant proteins and our bioassay expertise to validate that each lot of applicable InVivoPlusTM antibody binds strongly and specifically to its target antigen.” (https://bioxcell.com/what-we-do/)

Eukaryotic cell lines

Policy information about cell lines and Sex and Gender in Research

Cell line source(s)	THP-1 cell lines were provided by inflammasome expert Dr. Amal Amer, OSU Department of Microbial Infection and Immunity and were derived from THP-1 cells from the ATCC (ref TIB-202, established from a human male).<br>MDCK cells were received from BEI Resources (ref NR-2628, established from canine female).
Authentication	Cells from BEI resources were used at a low pass number from original, frozen stocks. GSDMD knockdown THP-1 cell lines were validated by Western blot analysis.
Mycoplasma contamination	All cells were treated for at least one week with mycoplasma removal reagent upon thawing. They were also routinely spot checked with Lonza MycoAlert detection reagent and confirmed to be mycoplasma negative.
Commonly misidentified lines<br>(See ICLAC register)	No commonly misidentified cell lines were used in this study.

Animals and other research organisms

Policy information about studies involving animals; ARRIVE guidelines recommended for reporting animal research, and Sex and Gender in Research

Laboratory animals	C57BL/6J (strain # 000664) and C57BL/6J-Gsdmdem1Vnce/J (Strain # 032663) used in this manuscript were purchased from Jackson Laboratories. No in-house breeding of these mice was performed. B6.Cg-Gt(ROSA)26Sortm14(CAG-tdTomato)Hze/J were purchased from Jackson Laboratories (strain #007914) and bred in-house. All mice were 8-12 weeks of age at the time of experimentation. Mice were housed in the Ohio State University's Biomedical Research Tower vivarium, which is maintained at 72+/-4 degrees F, with a 12:12 light dark cycle, and humidity between 30-70%. Autoclaved individually ventilated cages (Allentown) were used for housing. Mice were fed an irradiated natural ingredient chow diet ad libitum (Evnigo Teklad Diet 7912). Reverse osmosis purified water was provided through an automated rack water system. Cages included 1⁄4" corn cob bedding (Bed-o-Cobs, The Andersons) with cotton square nesting material.
Wild animals	No wild animals were used in the study.
Reporting on sex	Primary phenotypes were confirmed in male and female mice. Female mice were primarily used in mechanistic studies because they experience more severe influenza virus infections than their male counterparts as published and seen in our studies. Female mice provided a stringent model for exploring the effects of GSDMD and neutrophils in severe influenza virus infection.
Field-collected samples	No field-collected samples were used in the study.
Ethics oversight	All experiments were approved by The Ohio State University IACUC under protocol #2016A00000051-R2. This protocol adheres to the guidelines set out by the NIH adopted Guide for the Care and Use of Laboratory Animals.

Note that full information on the approval of the study protocol must also be provided in the manuscript.

Flow Cytometry

Plots

Confirm that:

- [x] The axis labels state the marker and fluorochrome used (e.g. CD4-FITC).
- [x] The axis scales are clearly visible. Include numbers along axes only for bottom left plot of group (a 'group' is an analysis of identical markers).
- [x] All plots are contour plots with outliers or pseudocolor plots.
- [x] A numerical value for number of cells or percentage (with statistics) is provided.

Methodology

Sample preparation	Lungs were harvested and single cell suspensions were created by enzymating digestion using type IV collagenase, DNase, and GentleMacs processing.
Instrument	CYTEK Aurora Flow Cytometer.
Software	FlowJo (v10.8.0) was used for flow cytometric data analysis.
Cell population abundance	Gating strategies are provided and no unusual or rare cell populations are studied in this manuscript.
Gating strategy	The entire gating strategy for all cell populations of interest are shown in Supplementary Figure 4.

- [x] Tick this box to confirm that a figure exemplifying the gating strategy is provided in the Supplementary Information.
